# Machine learning identifies neutrophil extracellular traps-related biomarkers for acute ischemic stroke diagnosis

**DOI:** 10.3389/fneur.2025.1611776

**Published:** 2025-08-29

**Authors:** Haipeng Zhang, Ti Wu, Xinghua Li, Shuangqing Liu, Yuanyuan Wang, Yang Cao

**Affiliations:** ^1^Department of Clinical Laboratory, The Second Hospital of Tianjin Medical University, Tianjin, China; ^2^Department of Neurology, Tianjin Medical University General Hospital, Tianjin, China; ^3^Department of Medical Laboratory, Fenyang College of Shanxi Medical University, Fenyang, China

**Keywords:** ischemic stroke, neutrophil extracellular traps, bioinformatics analysis, biomarker, machine learning

## Abstract

**Purpose:**

This study aimed to investigate the diagnostic potential of neutrophil extracellular traps (NETs)-related genes in acute ischemic stroke (AIS) through comprehensive bioinformatics analysis.

**Methods:**

Two GEO datasets (GSE37587 and GSE16561) were integrated to identify differentially expressed genes (DEGs) between AIS patients and healthy controls. Gene Set Enrichment Analysis (GSEA) was performed to explore functional pathways, while single-sample GSEA (ssGSEA) was used to evaluate immune cell infiltration patterns. NETs-related DEGs (NDEGs) were identified by intersecting the DEGs with previously reported NETS-related genes. Functional enrichment of NDEGs was performed using Gene ontology (GO) and Kyoto Encyclopedia of Genes and Genomes (KEGG) analyses. Key genes were identified via machine learning algorithms, including least absolute shrinkage and selection operator (LASSO) and random forest (RF). A diagnostic model was constructed based on the identified hub genes and validated using an independent dataset (GSE58294). Potential regulatory miRNAs and candidate therapeutic compounds were predicted using the TargetScan and DSigDB databases, respectively.

**Results:**

The discovery dataset included 73 AIS patients and 24 healthy controls, revealed 551 DEGs (225 upregulated, 326 downregulated). The analysis of ssGSEA revealed notable immune dysregulation in AIS patients, characterized by increased neutrophil infiltration and decreased level of Th17, Th1, and TFH cells. GSEA indicated that DEGs were enriched in neutrophil degranulation and innate immune system. NDEGs were significantly enriched in immune regulation and leukocyte apoptosis (GO) and NETs formation pathway (KEGG). Four hub genes—*SRC, TLR8, FCAR*, and *HIF1A*—were identified using LASSO and RF algorithms. A diagnostic model based on these genes yielded area under the curve (AUC) values of 0.880 in the training dataset and 0.936 in the validation dataset. Furthermore, three regulatory miRNAs (miR-146a-5p, miR-155-5p, and miR-21-5p) and 23 candidate therapeutic drugs were predicted.

**Conclusion:**

To our knowledge, this represents the first comprehensive investigation of NETs-related gene signatures in AIS patients compared with healthy controls. These findings deepen our understanding of immune cell infiltration and the underlying molecular mechanisms involved in stroke, offering novel insights that may enhance diagnostic accuracy and therapeutic strategies for AIS.

## Introduction

Ischemic stroke (IS) is a neurological disorder characterized by an inadequate supply of blood and oxygen to brain tissue, resulting from the obstruction or narrowing of cerebral blood vessels (1). As a major global health burden, stroke affects approximately 26 million individuals annually, making it the second leading cause of mortality worldwide (2). Among the various types of cerebrovascular diseases, ischemic stroke is the most prevalent, accounting for nearly 67% of all stroke cases (3). Acute ischemic stroke (AIS) accounts for approximately 70% of all newly diagnosed stroke (4). Individuals with a history of stroke face a significantly elevated risk of recurrence, long-term disability, and mortality (5). At present, the diagnosis of IS relies primarily on clinical evaluation and neuroimaging techniques (6). However, the absence of reliable blood-based biomarkers remains a major challenge, particularly in cases with atypical symptoms or inconclusive imaging findings (7, 8).

Neutrophil extracellular traps (NETs) are web-like extracellular structures released by neutrophils in response to various stimuli, including bacteria, fungi, inflammatory factors, chemokines, and activated platelets (9). Emerging research suggests that NETs play a critical role in the pathophysiology of stroke by exacerbating blood-brain barrier disruption, promoting thrombosis, inducing resistance to fibrinolytic agents, damaging vascular structures, and participating in revascularization processes (10). Furthermore, bioinformatics studies have identified the diagnostic potential of NETs-related genes (NRGs) in ischemia/reperfusion injury (11). Nevertheless, comprehensive investigations into the relationship between IS and NRGs remain limited.

To address this knowledge gap, we conducted a comprehensive bioinformatics analysis to elucidate the role of neutrophil extracellular traps (NETs) in ischemic stroke (IS), focusing on their associated genes and biological functions. Our study not only advances the understanding of the NETs-IS relationship but also investigates the mechanistic basis of IS pathogenesis. Furthermore, by identifying 4 key hub genes, we developed a predictive model for IS and identified potential regulatory miRNAs and therapeutic agents targeting these genes. These findings provide a foundation for future mechanistic studies and offer promising translational applications for stroke management.

## Methods

### Data selection and preparation

Gene expression profiles were retrieved from the GEO database^1^ using the search term “ischemic stroke.” Candidate datasets were screened according to the following criteria: (1) expression data derived from microarray platforms; (2) inclusion of at least 20 IS patients per dataset; and (3) whole blood samples collected within 48 h of symptom onset. For individuals with multiple blood collection time points, only the most recent sample was included in the analysis. Based on these criteria, three datasets were selected: GSE37587 (34 IS patients), GSE16561 (39 IS patients and 24 healthy controls), and GSE58294 (23 IS patients and 23 healthy controls). All subsequent bioinformatics analyses were conducted using R software (version 4.4.2).

The discovery dataset consisted of two gene expression datasets—GSE37587 and GSE16561—which were merged based on their shared microarray platform (GPL6883). The third dataset, GSE58294, was used as an independent validation dataset. Raw data underwent preprocessing, including background correction, log_2_ transformation, and normalization using the “limma” package (10). To mitigate batch effects between the merged datasets, we employed the ComBat algorithm from the “sva” package (11). Differentially expressed genes (DEGs) between IS patients and controls were identified using the “limma” package, with significance thresholds set at an adjusted *p*-value < 0.05 (Benjamini–Hochberg false discovery rate) and an absolute log_2_ fold change (|logFC|) > 0.05.

### Immune cell infiltration analysis

Single-sample Gene Set Enrichment Analysis (ssGSEA) was conducted to quantify the relative abundance of 24 infiltrating immune cell types, using a signature set derived from published literature (12). The analysis was performed using the R package “gsva” (13). For comparisons of immune cell enrichment scores between groups, the Wilcoxon rank-sum test was applied. Differential patterns of immune cell infiltration between the stroke and control cohorts were visualized using the R packages “ggplot2” and “ComplexHeatmap.

### NDEGs identification and functional enrichment analysis

NETs-related genes (NRGs) were obtained from a previously published study (14). To identify NETs-related differentially expressed genes (NDEGs), we intersected the differentially expressed genes (DEGs) with the NRGs. The resulting NDEGs were visualized using Venn diagrams, volcano plots, and heatmaps, generated using the R packages “VennDiagram,” “ggplot2,” and “pheatmap,” respectively. To explore the biological roles of the NDEGs, functional enrichment analysis was conducted, focusing on biological processes (BP), cellular components (CC), molecular functions (MF), and associated pathways. Gene Ontology (GO) and Kyoto Encyclopedia of Genes and Genomes (KEGG) pathway analyses were performed using the R package “clusterProfiler,” with *p* < 0.05 considered statistically significant (15). In addition, gene set enrichment analysis (GSEA) was performed based on the MSigDB database using the “clusterProfiler” package.

### Screening of key genes

To identify key genes among the NDEGs, machine learning approaches were applied. First, Least Absolute Shrinkage and Selection Operator (LASSO) regression was used to reduce dimensionality and select the most relevant genes (16). The LASSO model was optimized via a grid search with 10-fold cross-validation (CV) to identify the optimal regularization parameter, ensuring that the selected genes were strongly associated with the outcome while minimizing overfitting. Model performance was evaluated by calculating the mean squared error (MSE) across the cross-validation folds. Next, a Random Forest (RF) algorithm was applied to further refine gene selection based on importance scores (17). The RF model was optimized by performing a grid search with 5-fold cross-validation. Overfitting was mitigated by evaluating model performance on an independent validation dataset and monitoring the out-of-bag (OOB) error rate. The final gene selection was based on the importance ranking derived from the RF model. Both LASSO and RF analyses were implemented using the R packages “glmnet” and “randomForest,” respectively.

### Construction and validation of the diagnostic model

A diagnostic model for ischemic stroke was constructed using logistic regression based on the selected key genes, implemented via the R package “rms.” The model’s performance was assessed by receiver operating characteristic (ROC) curve analysis using the “pROC” package, with the area under the curve (AUC) calculated to evaluate diagnostic accuracy. Differential expression of the key genes and the diagnostic efficacy were further validated using the GSE58294 dataset.

### Prediction of pivotal miRNAs and candidate drugs

To explore the regulatory mechanisms of the identified key genes, pivotal miRNAs targeting these genes were predicted using the TargetScan database. In parallel, candidate drugs potentially capable of modulating the expression of these genes were identified using DSigDB database. Both analyses were conducted via the Enrichr platform.^2^ Candidate miRNAs and drugs were screened using a significance threshold of *p* < 0.05. All resulting networks were visualized using Cytoscape software (version 3.10.3).

## Results

### DRGs between stroke patients and healthy controls

**Figure 1:**
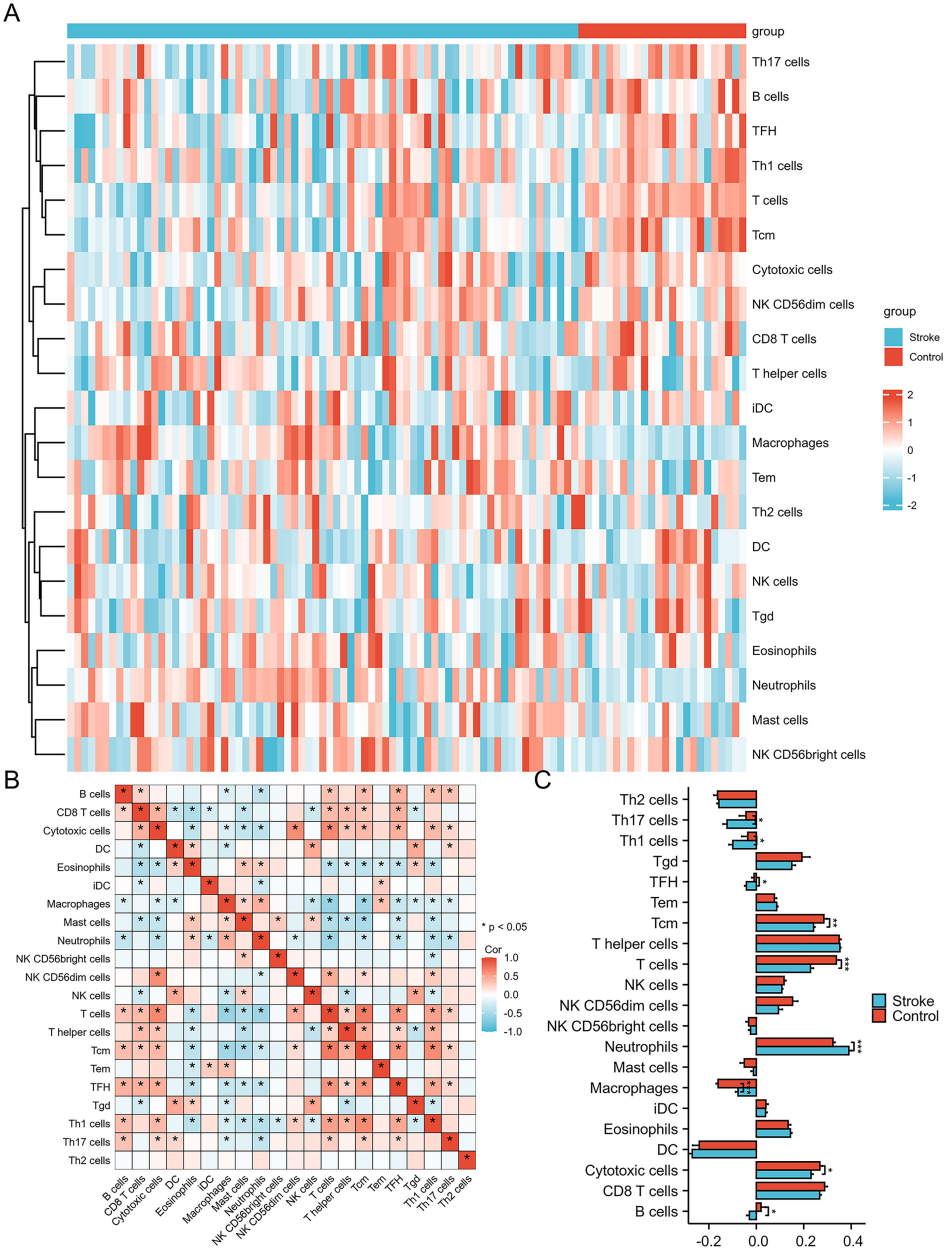
Differentially infiltrated immune cells in IS patients and healthy controls. **(A)** Heatmap of differential immune cells. **(B)** Correlation matrix of immune cells. **(C)** Histogram of differential immune cells.

The merged discovery dataset included 73 ischemic stroke patients and 24 healthy controls, identifying 225 upregulated genes and 326 downregulated genes. Principal component analysis (PCA) demonstrated that the two original datasets, which were initially distributed in distinct clusters, were successfully integrated into a unified distribution following batch effect correction (Supplementary Figures S1A,B). Box plots further confirmed consistent normalization across datasets (Supplementary Figures S1C,D). Single-sample Gene Set Enrichment Analysis (ssGSEA) was performed to assess immune cell infiltration, revealing a significant increase in neutrophil levels and a decrease in Th17, Th1, and T follicular helper (TFH) cells in stroke patients compared to healthy controls. Correlation analysis across 21 immune cell types demonstrated that neutrophils were positively associated with eosinophils and macrophages, but strongly negatively correlated with B cells, cytotoxic cells, T cells, and Th1 cells (Figure 1).

### Identification of NDEGs

**Figure 2:**
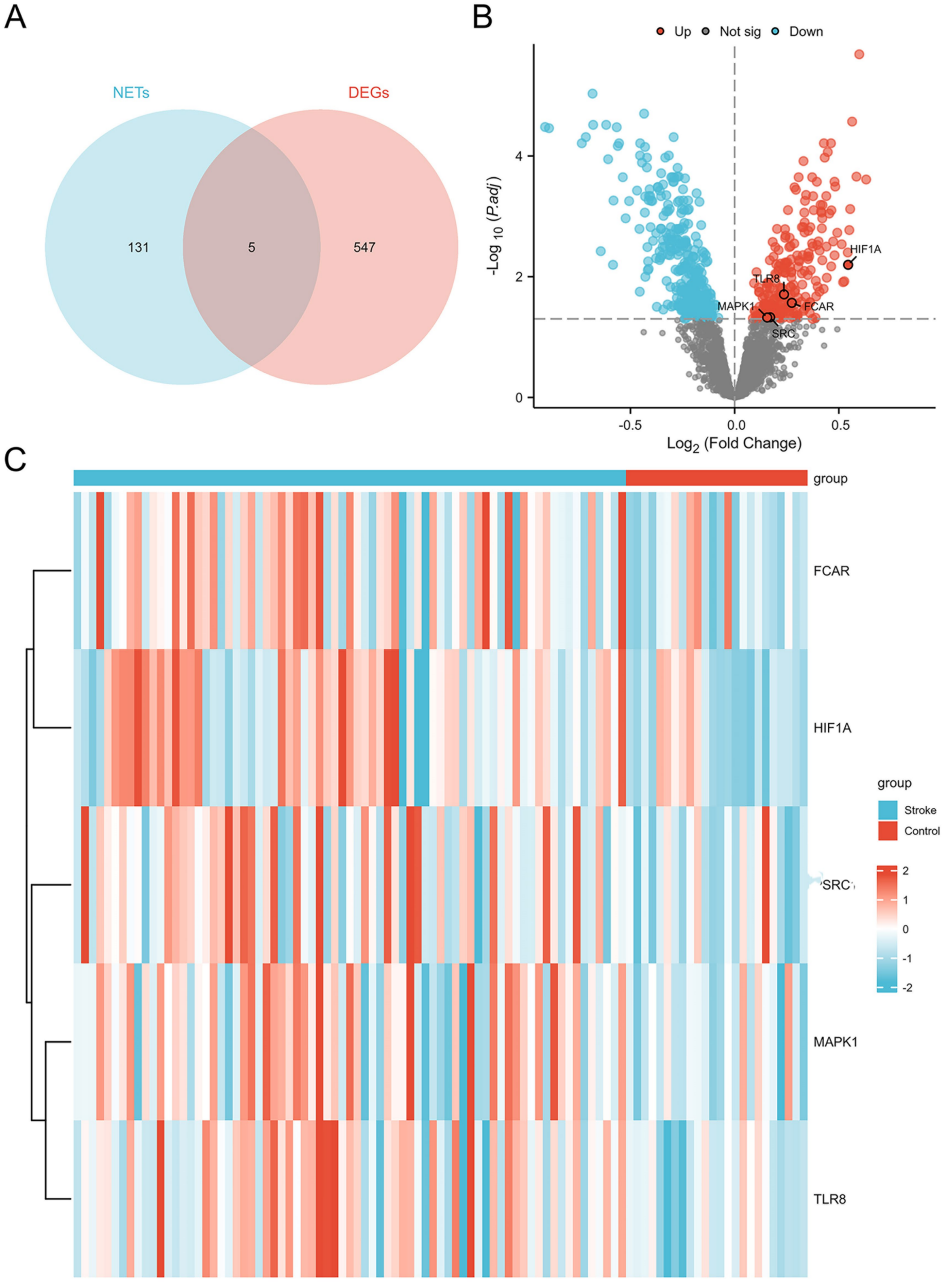
Identification of NDEGs. **(A)** Venn diagram of the intersection of NETS and DEGs. **(B)** Volcano plots of DE-GS distribution. **(C)** Heatmap of NDEGs distribution.

NRGs were obtained from a previously published study, with details provided in Supplementary Table S1. Intersection of NRGs with DEGs yielded five NDEGs: *SRC, TLR8, FCAR, HIF1A,* and *MAPK1*. All these five genes were significantly upregulated in ischemic stroke patients compared to healthy controls, as illustrated in the volcano plot and heatmap (Figure 2).

### Functional enrichment analysis

**Figure 3:**
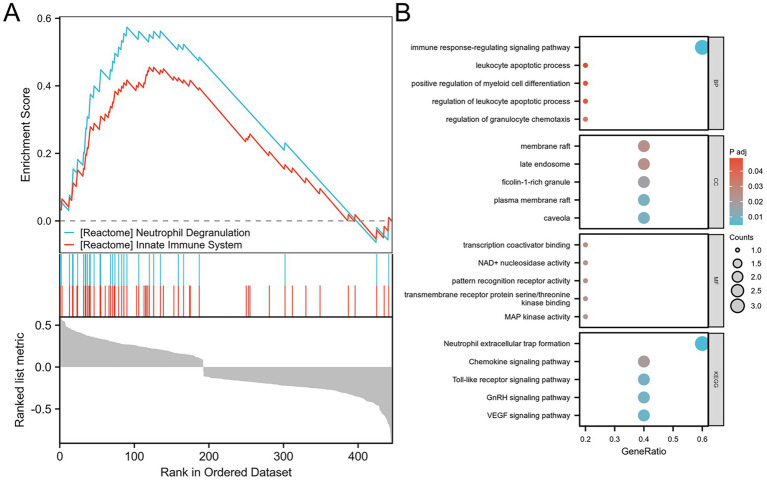
Functional enrichment analysis. **(A)** GSEA analysis of DEGs between stroke patients and healthy controls. **(B)** GO/KEGG analysis of differential NDEGs.

Gene Set Enrichment Analysis (GSEA) of the DEGs revealed significant enrichment in pathways related to neutrophil degranulation and the innate immune system (Figure 3A). After identifying the NDEGs, GO and KEGG enrichment analyses were conducted to explore their biological roles. GO results indicated that these NDEGs were primarily involved in immune response regulation and leukocyte apoptosis. KEGG pathway analysis further revealed that the NDEGs were significantly enriched in the pathway associated with NETs formation (Figure 3B). In conclusion, these findings highlight the pivotal role of inflammation and neutrophil-mediated immune responses—particularly NETs—in the pathogenesis of ischemic stroke.

### Identification of key genes and construction of the diagnostic model

**Figure 4:**
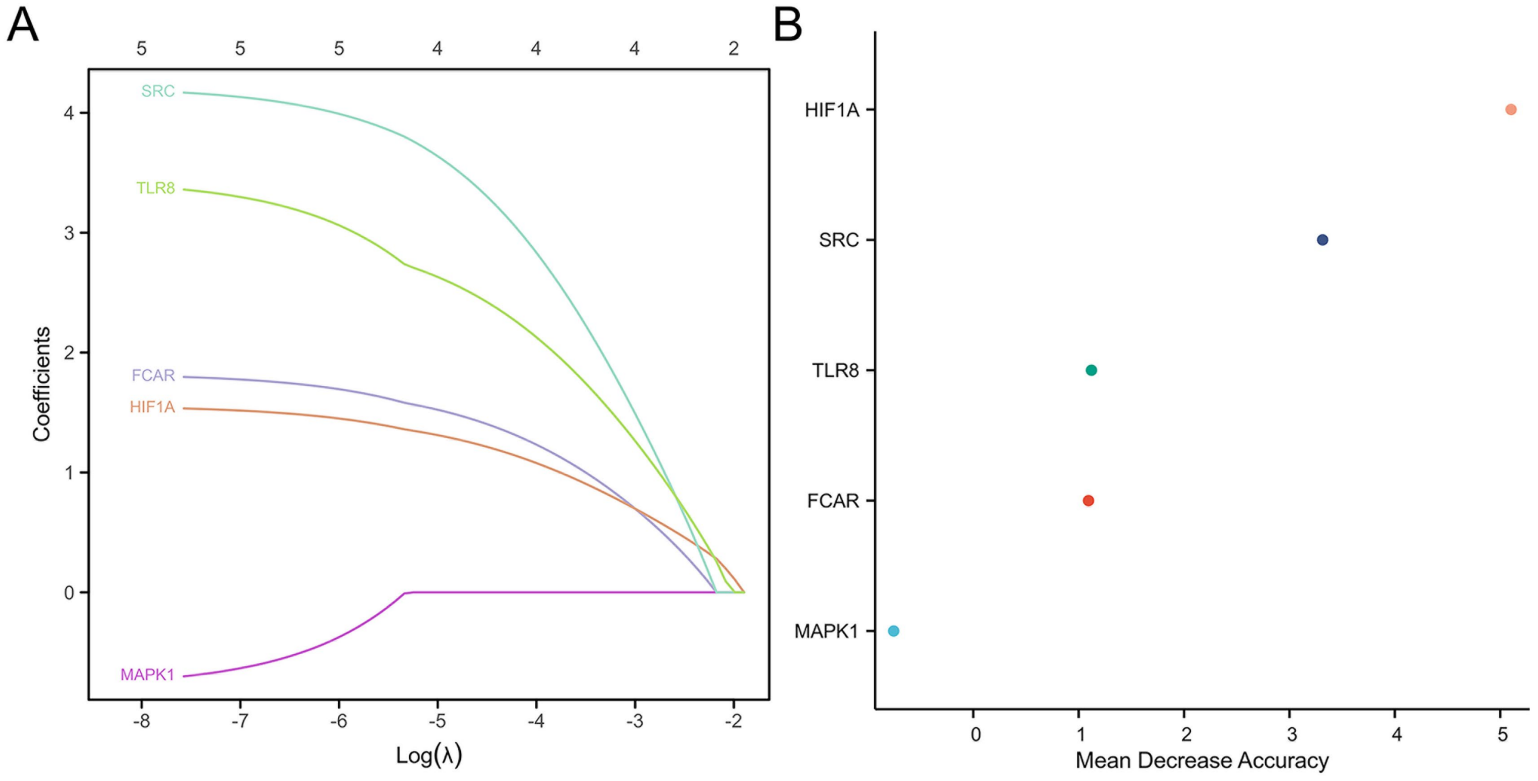
Screening for potential diagnostic biomarkers by machine learning. **(A)** LASSO. **(B)** Random forest.

**Figure 5:**
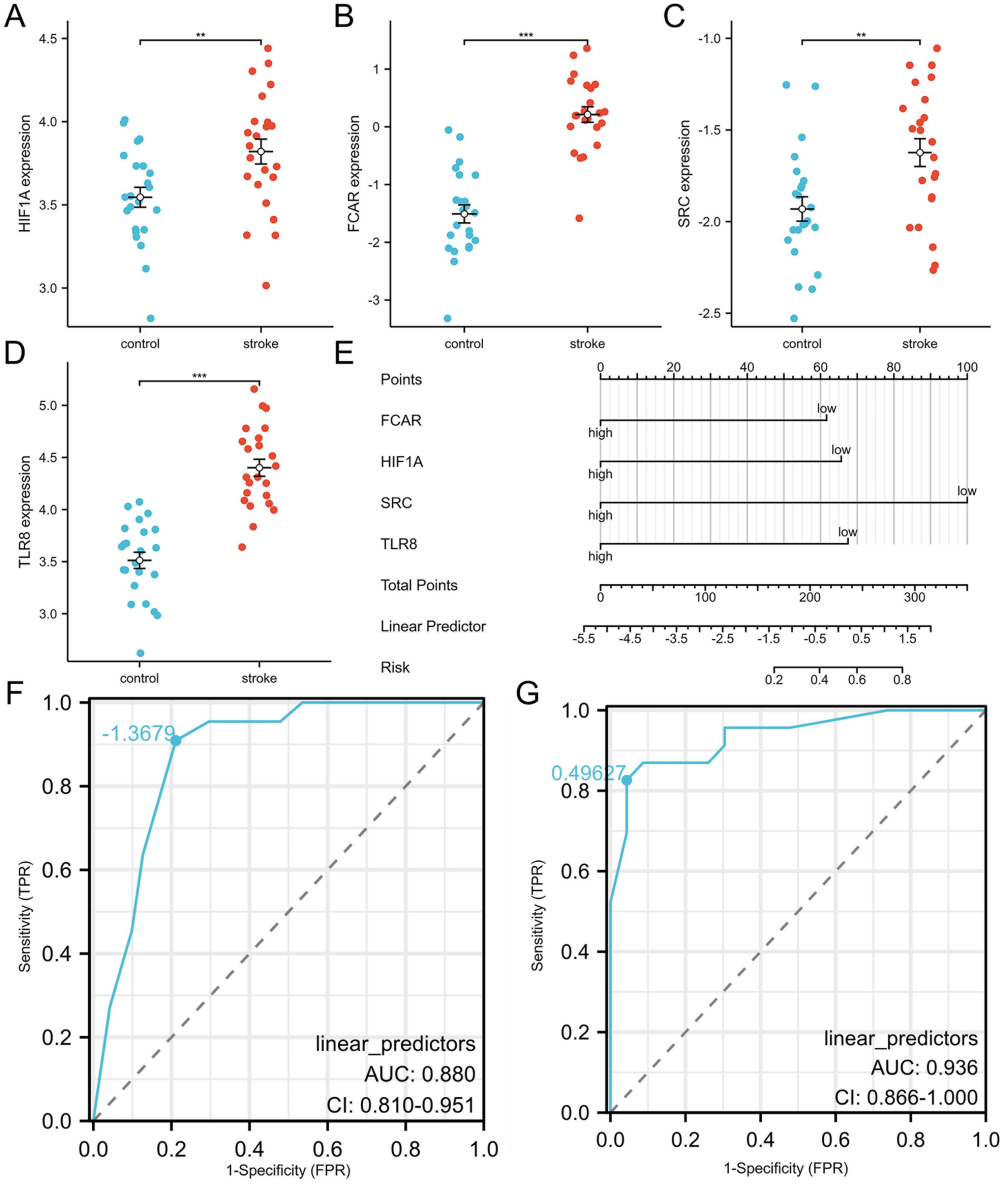
Diagnostic model construction and validation. **(A–D)** HIFIA, FCAR, SRC and TLR8 expression levels in validation dataset. **(E)** Construction of a nomogram model with 4 feature genes. **(F,G)** ROC curve for evaluating and validating the diagnostic model’s performance.

Using LASSO regression, the initial set of NDEGs was narrowed down to four diagnostic biomarkers: *SRC, TLR8, FCAR,* and *HIF1A* (Figure 4A). These genes were further validated using RF algorithms, which confirmed their importance based on feature rankings (Figure 4B). A nomogram incorporating the four key genes was then developed to predict the likelihood of ischemic stroke (Figure 5E). The model’s diagnostic performance was assessed using ROC curve analysis, resulting in an AUC of 0.880, indicating high predictive accuracy for ischemic stroke diagnosis (Figure 5F).

### Validation of key genes

To confirm the reliability and generalizability of the diagnostic model, the expression levels of the four key genes were validated in the independent dataset GSE58294 (Figures 5A–D). All four biomarkers (*SRC, TLR8, FCAR,* and *HIF1A*) were significantly upregulated in ischemic stroke patients compared to healthy controls. The diagnostic model’s performance was further validated in this external cohort, achieving an AUC of 0.936 in ROC analysis, which demonstrates the model’s robust predictive capability (Figure 5G).

### Identification of pivotal miRNAs and candidate drugs

Potential miRNAs targeting the four key genes were predicted using the TargetScan database, applying a significance threshold of *p* < 0.05. The analysis revealed that *SRC* was targeted by three miRNAs: hsa-miR-4669, hsa-miR-3619-3P, and hsa-miR-4537. *FCAR* was predicted to interact with hsa-miR-4669 and hsa-miR-4537, while *TLR8* was associated solely with hsa-miR-3619-3P.

**Figure 6:**
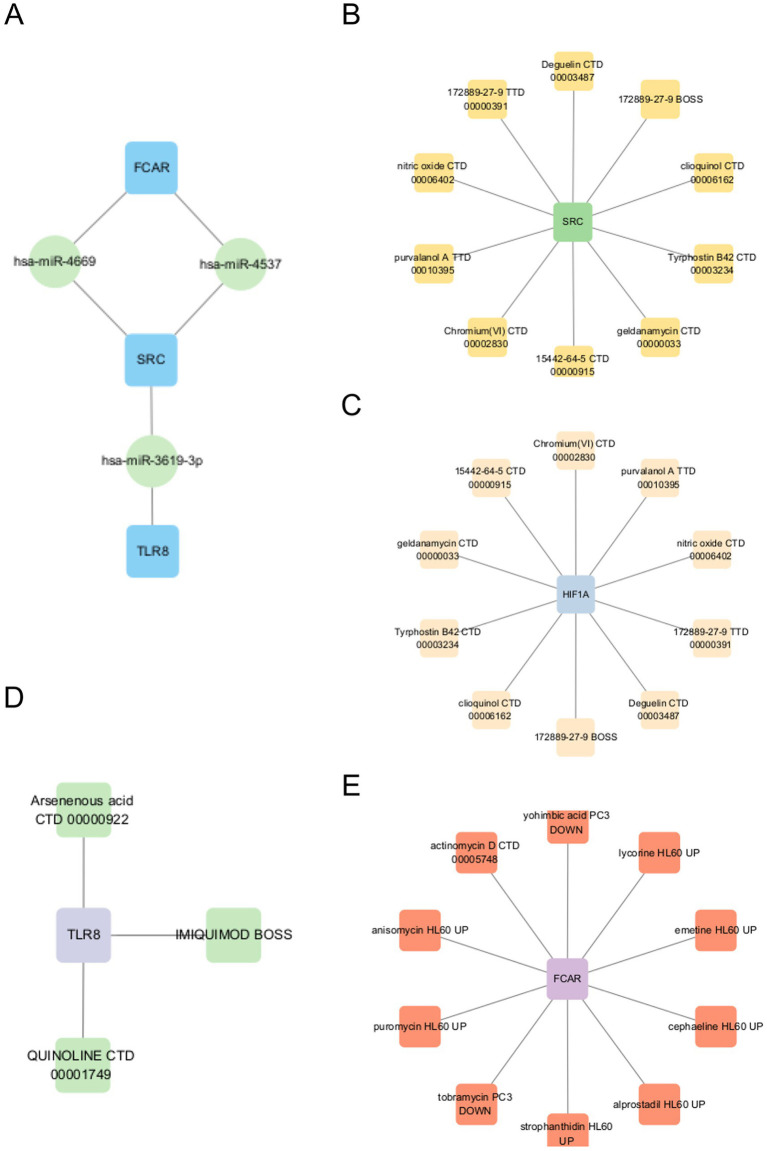
**(A)** Potential miRNAs targeting 4 key genes. **(B–E)** candidate drugs targeting 4 key genes.

Additionally, candidate drugs modulating the expression of the key genes were identified using the DSigDB database. The top 10 drug candidates for each gene were selected based on a significance threshold of *p* < 0.05. Notably, *SRC* and *HIF1A* shared the same top 10 candidate compounds, among which “172889-27-9 TTD 00000391” had the highest combined score (17891). For *FCAR*, actinomycin D (CTD 00005748) yielded the highest score (794). In contrast, only three drug candidates (Arsenenous acid CTD 00000922, IMIQUIMOD BOSS and QUINOLINE CTD 00001749) were identified for *TLR8*. These findings offer novel insights into the post-transcriptional regulation and therapeutic targeting of ischemic stroke (Figure 6).

## Discussion

To our knowledge, this represents the first comprehensive investigation of NETs-related gene signatures in AIS patients compared with healthy controls. Our findings demonstrated that NETs-related genes are involved in the immune response processes underlying AIS. Using machine learning approaches, we identified four key genes—*SRC, TLR8, FCAR,* and *HIF1A*—that exhibit strong diagnostic potential. A diagnostic model based on these genes showed high predictive accuracy, and subsequent analyses identified putative miRNAs and candidate drugs targeting these genes. Collectively, these findings provide novel insights that may contribute to the advancement of diagnostic and therapeutic strategies for AIS.

Following stroke onset, neutrophils are the earliest immune cells to become activated and infiltrate ischemic brain tissue, with their numbers increasing markedly within hours (18). Activated neutrophils undergo morphological changes, including chromatin condensation, nuclear membrane fragmentation, and the fusion of nucleoplasm with chromatin. Eventually, the cells condense into a spherical shape, and the cell membrane ruptures, releasing intracellular components and forming fibrous structures known as NETs (19). In 2015, Perez-de-Puig et al. first reported histone citrullination in neutrophils of stroke patients, accompanied by extracellular release of DNA and histones (20). Two years later, Laridan et al. further confirmed the critical role of NETs in thrombus formation in patients with stroke (21). In the same year, Valles et al. identified significantly elevated plasma levels of NETs markers—including citrullinated histone 3 (CitH3), cell-free DNA (cfDNA), and nucleosomes—in stroke patients, with increases of 72, 33, and 39%, respectively, compared to healthy controls (22). NETs levels begin to rise within 24 h of stroke onset, peaking around 2–3 days, and remaining elevated for up to 7 days. Accumulating evidence suggests that NETs contribute to the stroke pathology through multiple mechanisms: (1) accelerating blood–brain barrier disruption; (2) promoting thrombus formation; (3) inducing resistance to tissue plasminogen activator (t-PA); and (4) impairing vascular integrity and inhibiting remodeling (10). These findings underscore the central role of NETs in the progression of AIS and support the relevance of NETs-related genes as potential diagnostic and therapeutic targets.

In this study, we identified 551 DEGs from a combined dataset comprising 73 ischemic stroke patients and 24 healthy controls. Immune cell infiltration analysis revealed a notable trend: a significant increase in neutrophil infiltration accompanied by a reduction in lymphocyte populations, including both T cells and B cells. Although neutrophils and lymphocytes are both integral components of the immune response, they displayed markedly divergent patterns in stroke patients. This imbalance may be attributed to the massive infiltration of neutrophils into ischemic brain tissue, which disrupts neuroimmune homeostasis and induces a shift in the peripheral immune system toward a suppressed state (23). This immune suppression is characterized by reduced lymphocyte counts and impaired lymphocyte function (24–26), and increases susceptibility to systemic infections, supporting our earlier hypothesis that neutrophil levels are positively correlated with infection risk in stroke patients (27). By intersecting DEGs with previously reported NETs-related genes, we identified five key genes: *SRC, TLR8, FCAR, MAPK1* and *HIF1A*. Functional enrichment analysis revealed that these genes are significantly involved in immune regulatory pathways and leukocyte apoptosis, reinforcing their connection to NETs-mediated immune dysregulation in stroke. These findings underscore the central role of NETs-related genes in the pathophysiology of stroke and suggest that they may serve as promising diagnostic and therapeutic targets.

Based on the four key genes identified through the machine learning, we constructed a diagnostic nomogram, which demonstrated high predictive performance, with AUC values of 0.880 in the training dataset and 0.936 in the validation dataset. Most of the key genes has been implicated in stroke-related pathogenesis. *SRC*, a non-receptor protein tyrosine kinase encoded by *SRC* proto-oncogene, exhibits rapid activation within hours following stroke onset, with expression levels progressively increasing in infarcted regions for up to 24 h (28). It exacerbates stroke damage by promoting M1 microglial polarization, enhancing inflammation, and inducing neuronal apoptosis (29). Toll-like receptor (TLR) are transmembrane pattern recognition receptors encoded by *TLR* genes, which initiate intracellular signaling cascades upon recognition of pathogen-associated molecular patterns (PAMPs) (30). In AIS, TLR8 exacerbates neuronal damage by promoting apoptosis, mediating T cell-driven inflammation, and has been proposed as a biomarker of poor prognosis (31, 32). *HIF1A*, a master regulator of cellular oxygen homeostasis, modulates the expression of genes involved in hypoxic adaptation (33). In the context of AIS, it contributes to inflammasome activation, mitochondrial dysfunction, and cell death under severe hypoxia (34).

Finally, we predicted miRNAs and candidate drugs that may regulate the identified key genes. Among them, miR-4669 and miR-4537 were found to simultaneously target *FCAR* and *SRC*, while miR-3619-3P was predicted to regulate *TLR8* and *SRC*. Although the roles of these miRNAs in AIS remain unclear, studies in other disease contexts provide preliminary insights. miR-4669 has been reported to enhance tumor invasiveness and contribute to an immunosuppressive tumor microenvironment (35). miR-4537 inhibits tumor cell proliferation, promotes apoptosis, and increases cellular radiosensitivity (36). In contrast, miR-3619-3P facilitates tumor cell migration and invasion (37). These findings suggest that these miRNAs may participate in the regulation of immune and inflammatory pathways in AIS, but their specific functions in stroke pathogenesis warrant further investigation.

PP2, a reversible ATP-competitive inhibitor of the Src family kinases (SFKs), demonstrates dual-targeting activity against both *SRC* and *HIF1A*. In the context of stroke, PP2 has been shown to attenuate blood–brain barrier (BBB) disruption by suppressing Src kinase phosphorylation (38). Similarly, Purvalanol A, a selective cyclin-dependent kinase (CDK) inhibitor, was predicted to target both *SRC* and *HIF1A*, with a binding affinity score slightly lower than that of PP2 (14,316 vs. 17,891). Emerging evidence suggests that Purvalanol A reduces ischemia/reperfusion-induced Golgi fragmentation and apoptosis through CDK inhibition (39). Nevertheless, the precise molecular mechanisms and therapeutic efficacy of Purvalanol A in stroke remain to be elucidated through further experimental studies.

The pathological progression of stroke is modulated by multiple regulatory systems, including the autonomic nervous system, hypothalamic–pituitary–adrenal (HPA) axis, and the immune system (40). We hypothesize that NETs may play a pivotal role in this process and directly contribute to stroke pathogenesis. However, the precise regulatory mechanisms underlying this involvement require further investigation. While our findings offer valuable insights, we acknowledge that the study is based entirely on in silico analyses, without experimental validation of the key findings. Moreover, the relatively small sample size and the absence of *in vivo* or *in vitro* validation of the predicted miRNAs and therapeutic candidates limit the generalizability and translational potential of our results. Future studies, including experimental validation (e.g., qPCR, Western blot, luciferase reporter assays), along with larger, well-characterized cohorts, are crucial for deepening our understanding of the relationship between these biomarkers and acute ischemic stroke. This will facilitate the development of more accurate diagnostic approaches and therapeutic interventions for stroke management.

## Data Availability

The original contributions presented in the study are included in the article/Supplementary material, further inquiries can be directed to the corresponding author.

## Footnotes

^1^ http://www.ncbi.nlm.nih.gov/geo

^2^ https://maayanlab.cloud/Enrichr/
