# Supplementary material for: Machine learning identifies neutrophil extracellular traps-related biomarkers for acute ischemic stroke diagnosis
Source: Front Neurol. 2025 Aug 29;16:1611776. doi: 10.3389/fneur.2025.1611776 (PMC12425765; doi:10.3389/fneur.2025.1611776)
Supplement: Supplementary file 2 [file Table_1.docx]

**Table S1:** Information of neutrophil extracellular traps related genes.

| **Gene symbol** | **Description** |
| --- | --- |
| ACTB | Actin beta |
| ACTG1 | Actin gamma 1 |
| ACTN1 | Actinin alpha 1 |
| ACTN4 | Actinin alpha 4 |
| AKT1 | AKT serine/threonine kinase 1 |
| AKT2 | AKT serine/threonine kinase 2 |
| ARPIN | Actin related protein 2/3 complex inhibitor |
| ATG7 | Autophagy related 7 |
| AZU1 | Azurocidin 1 |
| C3 | Complement C3 |
| C3AR1 | Complement C3a receptor 1 |
| C5AR1 | Complement C5a receptor 1 |
| CAMP | Cathelicidin antimicrobial peptide |
| CARD11 | Caspase recruitment domain family member 11 |
| CASP1 | Caspase 1 |
| CAT | Catalase |
| CCDC25 | Coiled-coil domain containing 25 |
| CCL2 | C-C motif chemokine ligand 2 |
| CCL3 | C-C motif chemokine ligand 3 |
| CCL4 | C-C motif chemokine ligand 4 |
| CCL5 | C-C motif chemokine ligand 5 |
| CD177 | CD177 molecule |
| CD274 | Programmed cell death 1 ligand 1 |
| CD44 | CD44 molecule (Indian blood group) |
| CEBPB | CCAAT enhancer binding protein beta |
| CFTR | ATP-binding cassette sub-family C, member 7 |
| CLEC4E | Macrophage-inducible C-type lectin |
| CLEC6A | Dectin-2 |
| CLEC7A | Dectin-1 |
| CSF3 | Granulocyte colony stimulating factor |
| CTSC | Cathepsin C |
| CTSG | Cathepsin G |
| CXCL1 | C-X-C motif chemokine ligand 1 |
| CXCL2 | C-X-C motif chemokine ligand 2 |
| CXCR4 | C-X-C motif chemokine receptor 4 |
| CYBB | NADPH oxidase |
| DEFA3 | Defensin alpha 3 |
| DNAJB1 | DnaJ heat shock protein family (Hsp40) member B1 |
| DNASE1 | Deoxyribonuclease I |
| ELANE | Neutrophil elastase |
| ENO1 | Enolase 1 |
| ENTPD4 | Ectonucleoside Triphosphate Diphosphohydrolase 4 |
| F2RL2 | Proteinase-activated receptor-3 |
| F3 | Coagulation Factor III, tissue factor |
| FCAR | Fc fragment of IgA receptor |
| FCGR2B | Fc gamma receptor IIb |
| FGL2 | Fibrinogen like 2 |
| GPBAR1 | G protein-coupled bile acid receptor 1 |
| GSDMD | Gasdermin D |
| H2AX | H2A histone family, member X |
| HIF1A | Hypoxia inducible factor 1 subunit alpha |
| HMGB1 | High mobility group box 1 |
| HRG | Histidine rich glycoprotein |
| IL12A | Interleukin 12A |
| IL17A | Interleukin 17 |
| IL1B | Interleukin 1 beta |
| IL1RL1 | Interleukin 1 receptor like 1 |
| IL33 | Interleukin 33 |
| IL36RN | Interleukin 36 receptor antagonist |
| IL5 | Interleukin 5 |
| IL6 | Interleukin 6 |
| IL8 | Interleukin 8 |
| ILK | Integrin linked kinase |
| IRAK4 | Interleukin 1 receptor associated kinase 4 |
| IRF1 | Interferon regulatory factor 1 |
| ITGAM | Complement component 3 receptor 3 subunit |
| ITGB1 | Integrin subunit beta 1 |
| ITGB2 | Complement component 3 receptor 3 and 4 subunit |
| KCNN3 | Potassium channel, calcium activated |
| KLF2 | KLF transcription factor 2 |
| KRT10 | Keratin 10 |
| LCP1 | Lymphocyte cytosolic protein 1 |
| LDLR | Low density lipoprotein receptor |
| LPAR3 | Lysophosphatidic acid receptor 3 |
| LTF | Lactotransferrin |
| LYZ | Lysozyme |
| MAPK1 | Mitogen-activated protein kinase 1 |
| MAPK14 | Mitogen-activated protein kinase 14 |
| MAPK3 | Mitogen-activated protein kinase 3 |
| MAPK7 | Mitogen-activated protein kinase 7 |
| MCOLN3 | Mucolipin TRP cation channel 3 |
| MFN1 | Mitofusin 1 |
| MFN2 | Mitofusin 2 |
| MIR146A | MicroRNA 146a |
| MIR21 | MicroRNA 21 |
| MIR223 | MicroRNA 223 |
| MMP9 | Matrix metallopeptidase 9 |
| MNDA | Myeloid cell nuclear differentiation antigen |
| MPO | Myeloperoxidase |
| MTOR | Mechanistic target of rapamycin kinase |
| MYD88 | MYD88 innate immune signal transduction adaptor |
| MYH9 | Myosin heavy chain 9 |
| NFE2L2 | NFE2 like bZIP transcription factor 2 |
| NFIL3 | Nuclear factor, interleukin 3 regulated |
| NFKBIA | NFKB inhibitor alpha |
| NLRP3 | NLR family pyrin domain containing 3 |
| NOX4 | NADPH oxidase 4 |
| OPA1 | OPA1 mitochondrial dynamin like GTPase |
| ORAI1 | ORAI calcium release-activated calcium modulator 1 |
| P2RX1 | Purinergic receptor P2X 1 |
| PADI4 | Peptidyl arginine deiminase 4 |
| PARVB | Parvin beta |
| PF4 | Chemokine (C-X-C motif) ligand 4 |
| PIK3CA | Phosphatidylinositol-4,5-bisphosphate 3-kinase |
| PKM | Pyruvate kinase M1/2 |
| PROCR | Protein C receptor |
| PRTN3 | Proteinase 3 |
| PTAFR | Platelet activation factor receptor |
| RIPK1 | Receptor interacting serine/threonine kinase 1 |
| RIPK3 | Receptor interacting serine/threonine kinase 3 |
| S100A12 | S100 calcium binding protein 2 |
| S100A8 | S100 calcium binding protein A8 |
| S100A9 | S100 calcium binding protein A9 |
| S1PR2 | Sphingosine-1-phosphate receptor 2 |
| SELP | P-selectin |
| SELPLG | P-selectin receptor |
| SGK1 | Serum/glucocorticoid regulated kinase 1 |
| SIGLEC14 | Sialic acid binding Ig like lectin 4 |
| SOCS3 | Suppressor of cytokine signaling 3 |
| SPP1 | Secreted phosphoprotein 1 |
| SRC | SRC proto-oncogene, non-receptor tyrosine kinase |
| STAT3 | Signal transducer and activator of transcription 3 |
| SUCNR1 | Succinate receptor 1 |
| SYK | Spleen associated tyrosine kinase |
| TICAM1 | TIR domain-containing adaptor-inducing interferon-beta |
| TIMP1 | TIMP metallopeptidase inhibitor 1 |
| TKT | Transketolase |
| TLR2 | Toll like receptor 2 |
| TLR4 | Toll like receptor 4 |
| TLR7 | Toll like receptor 7 |
| TLR8 | Toll like receptor 8 |
| TLR9 | Toll like receptor 9 |
| TNF | Tumor necrosis factor-alpha |
| TNFAIP3 | TNF alpha induced protein 3 |
| WASL | WASP like actin nucleation promoting factor |
| XIST | X inactive specific transcript |
